# Management Dilemma: Is Enucleation With Lymphadenectomy a Viable Choice for Large Pancreatic Neuroendocrine Tumors (p-NETs)?

**DOI:** 10.7759/cureus.42482

**Published:** 2023-07-26

**Authors:** Vinay Kumar, Neelesh Shrivastava, Suprateek Saha, Sonveer Gautam, Bharati Pandya

**Affiliations:** 1 Department of Surgical Oncology, All India Institute of Medical Sciences, Bhopal, IND; 2 Department of General Surgery, All India Institute of Medical Sciences, Bhopal, IND

**Keywords:** gastrinoma, pancreatico-duodenectomy (pd), distal pancreatectomy (dp), enucleation with lymphadenectomy (el), pancreatic neuroendocrine tumor (pnets)

## Abstract

Managing pancreatic neuroendocrine tumors (pNETs) has gradually taken a trend toward conservative management owing to its slow-growing and prolonged course. Though clear criteria exist regarding managing small tumors, the direction of a large tumor remains a dilemma. We present a case of a young 26-year-old lactating woman with a large 3.4 cm × 3.2 cm mass in the uncinate process, which is adjacent to the inferior vena cava (IVC) and has flimsy adhesions to the duodenum. She also had an enhancing adjacent lymph node measuring 1.2 cm × 0.7 cm, which showed enhancement with Ga-68 DOTANOC positron emission tomography/computed tomography (PET/CT) and raised serum gastrin levels. The dilemma was between pancreaticoduodenectomy (PD) or enucleation with lymphadenectomy (EL). Finally, EL was conducted. We discussed this case with relevant studies, which we had referred to because large-sized tumors are not usually enucleated.

## Introduction

Pancreatic neuroendocrine tumors (pNETs) constitute 3% to 5% of the total pancreatic tumors [1]. The incidence of pNETs is estimated to be one to five cases per million per year [2]. Nonfunctional pNETs, in particular, are increasing in incidence and are increasingly diagnosed in earlier stages of the disease. This early diagnosis is due to improved incidental detection in imaging studies performed for another reason [2]. Most pNETs are nonfunctioning, but they can secrete various hormones, resulting in unique clinical syndromes [2]. With the widespread use of high-quality, cross-sectional imaging, small sporadic incidentaloma is increasingly diagnosed [3]. These neoplasms can be classified based on functional status, either functional or nonfunctional type. Functional-type neoplasms account for 30% of all pNETs [1]. Surgery is the treatment of choice for this neoplasm. Two surgical modalities commonly used are pancreaticoduodenectomy (PD) and enucleation with lymphadenectomy (EL). It is generally accepted that the indications for enucleation are the diameter of the tumor <2 cm and the cancer is at least 3 mm away from the central pancreatic duct [1]. Compared to pancreatic adenocarcinoma, pNETs have a better prognosis after surgery [4]. pNETs have markedly heterogeneous clinical presentations like small-size or low-grade tumors that tend to display an indolent presentation with little tumor progression over time, just opposite to that of large or high-grade tumors that have more substantial metastatic potential, thereby compromising the long-term outcomes of patients [5]. Enucleation seems to be a preferred modality for small pancreatic tumors against standard surgical resection, particularly for tumors away from the pancreatic duct. The proportion of enucleation surgeries is increasing; therefore, there is a need to understand how this surgical modality compares with the standard surgical resection regarding clinical outcomes [6].

## Case presentation

A 26-year-old female homemaker presented intermittently to the Surgical Oncology outpatient department with complaints of recurrent episodes of epigastric discomfort, characterized by a burning sensation, and vomiting sour contents in the postprandial period for the past four months. The discomfort would subside when the patient consumed proton pump inhibitors, which she was accustomed to taking. She was a lactating mother with a one-year-old baby. There was no history of previous surgeries, addictions, or previous medical illnesses. Additionally, there was no indication of any familial syndromes, and she was not taking any medications.

**Figure 1:**
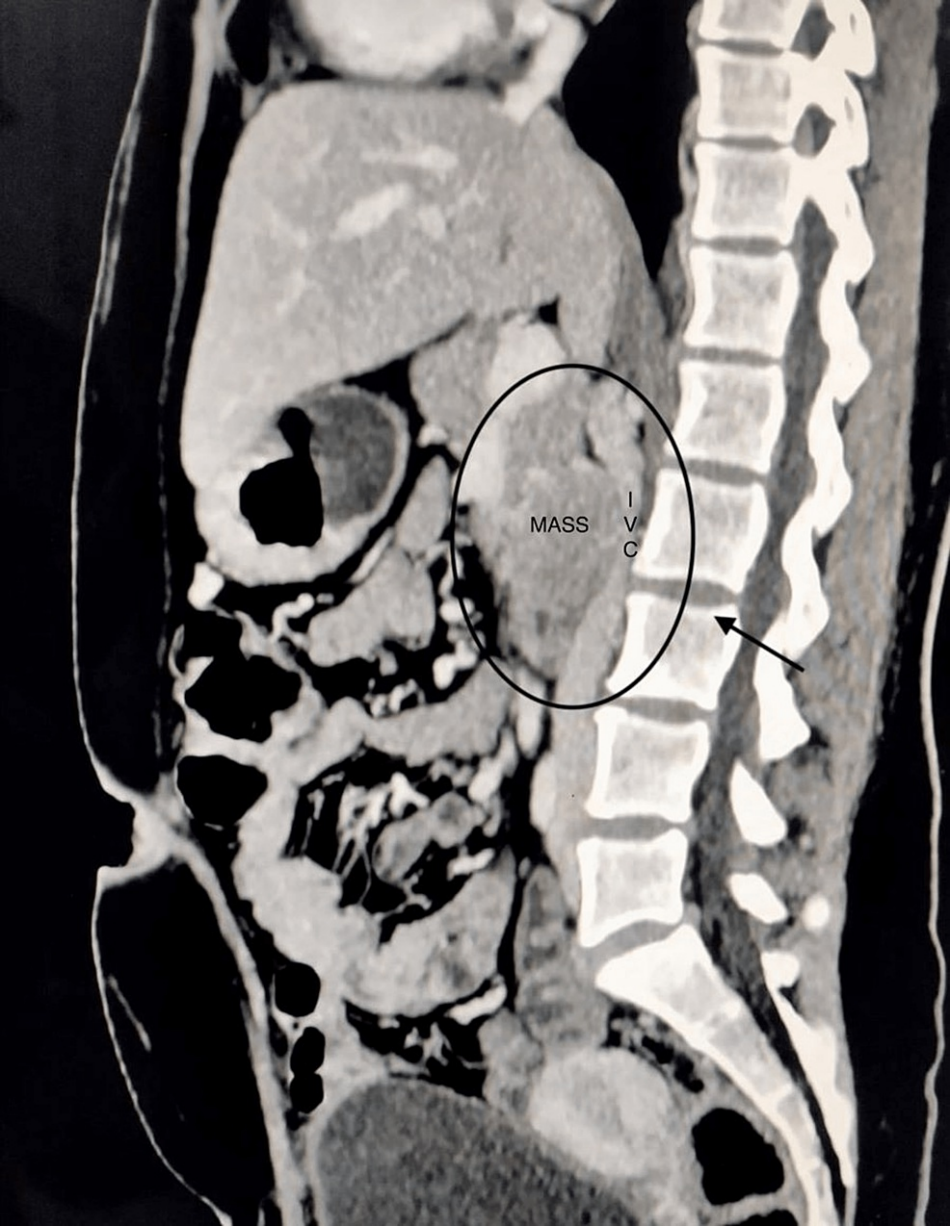
Sagittal section showing compression of the inferior vena cava posteriorly.

**Figure 2:**
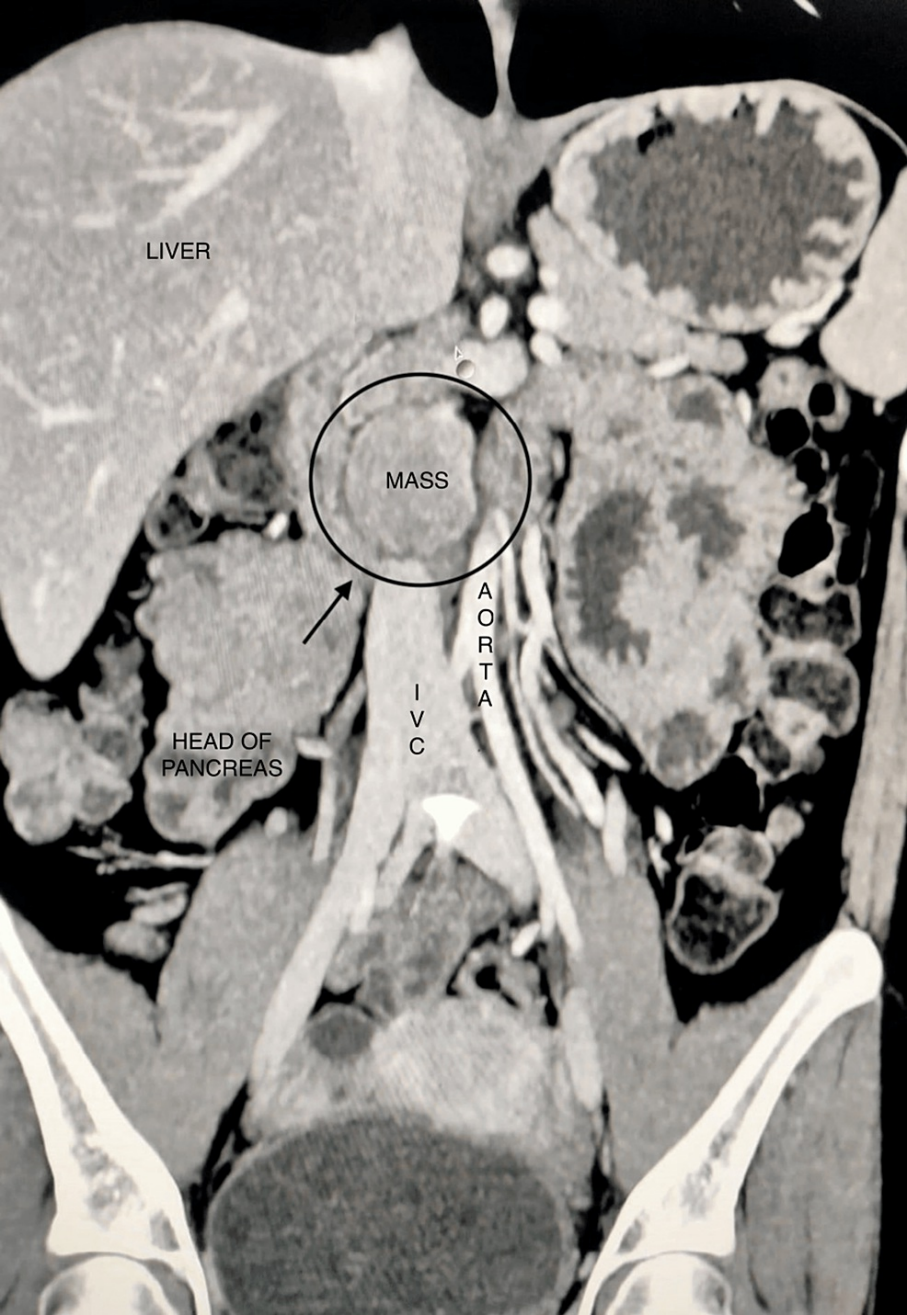
Coronal section scan images showing a large tumor arising from the uncinate process of the pancreas, compressing the inferior vena cava posteriorly and abutting the anterior aspect of the duodenum.

General and abdominal examinations revealed no significant findings. Upper gastrointestinal (GI) endoscopy revealed confluent linear erosion at the gastroesophageal junction. The stomach was normal, but the duodenum showed multiple erosions in the duodenal bulb, extending onto the second part of the duodenum. A biopsy was not taken. Specific blood tests were done, which revealed a serum gastrin level of 2,278 pg/mL (*N *< 100 pg/mL). A preoperative endocrine workup was conducted, revealing the following normal results: serum calcium was 8.4 mg/dL (normal range, 8-10.5 mg/dL), serum phosphorus 3.1 mg/dL (normal range, 3-4.5 mg/dL), serum albumin 3.8 mg/dL (normal range, 3.5-5 g/dL), and serum prolactin was 29.4 ng/dL (normal range, 2-30 ng/mL). Contrast-enhanced computed tomography (CECT) scan revealed a well-defined, smoothly margined soft tissue mass lesion measuring 34 mm × 32 mm in the precaval space, located posterior to the uncinate process of the pancreas. The mass was causing anterior displacement of the uncinate process and was positioned above the third part of the duodenum, exerting compression on the inferior vena cava (IVC, Figures 1-2).

**Figure 3:**
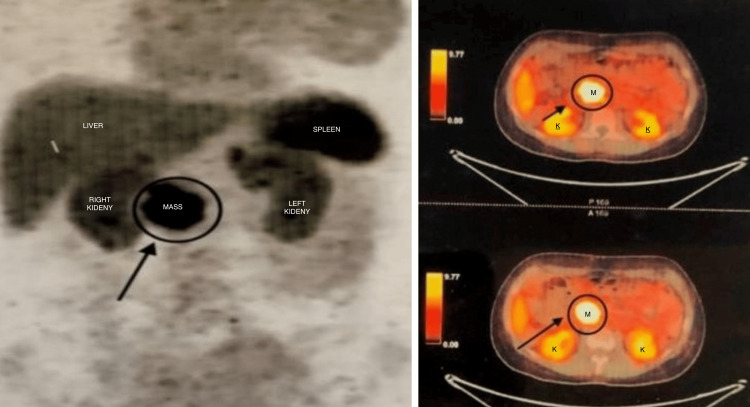
Three serial films of Ga-68 DOTANOC PET/CT uptake in a soft tissue mass (M) showing 3.4 cm × 3.2 cm and extending up to 4.7 cm in the pre-renal space (K = kidneys) posteriorly to the uncinate process with loss of fat planes with the IVC inferiorly and compressing it anteroposteriorly. PET CT films. IVC, inferior vena cava; PET, positron emission tomography; CT, computed tomography

A Ga-68 DOTANOC PET/CT scan was conducted, revealing an area of abnormal intensity with increased uptake in a soft tissue mass measuring 3.4 cm × 3.2 cm. The mass extended up to 4.7 cm in the prerenal space, located posterior to the uncinate process. The scan also showed the loss of fat planes with the IVC below the mass and compression of the IVC in the anteroposterior direction (Figure 3). In addition, a lymph node was observed anterosuperior to this lesion, measuring 1.2 x 0.7 cm in size with enhanced uptake.

**Figure 4:**
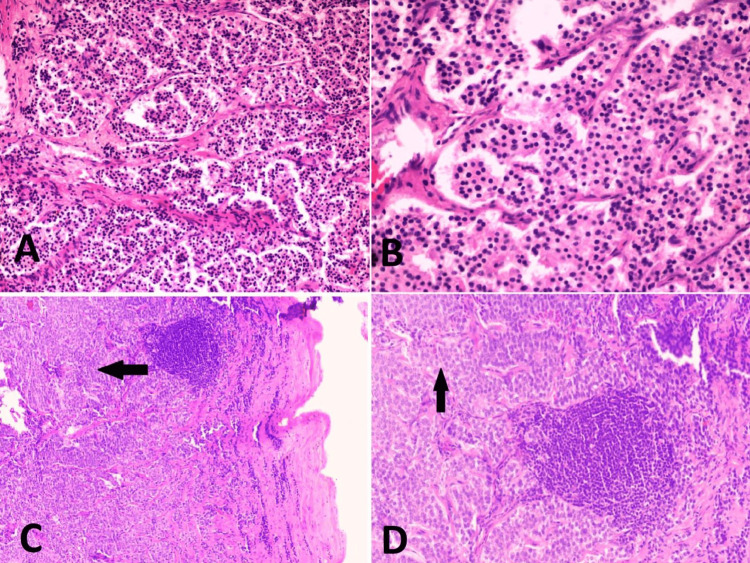
The features were consistent with a neoplastic pathology, suggestive of a neuroendocrine tumor. (A and B) Cells exhibited round nuclei with stippled chromatin, indistinct nucleoli, and a scant amount of finely granular eosinophilic cytoplasm. (C and D) The histopathology of the lymph nodes (post-op) revealed metastatic deposits of neuroendocrine tumors in lymph nodes.

CT-guided biopsy from the mass revealed a circumscribed tumor composed of uniform monomorphic rounded cells arranged in nests and organoid patterns separated by fibrovascular septa. The tumor cells showed round nuclei with stippled chromatin, indistinct nucleoli, and a scant amount of finely granular eosinophilic cytoplasm. Mitotic activity was inconspicuous (less than 2 per 10 high-power fields [HPFs]). No necrosis was seen. Features were consistent with a neoplastic pathology and were suggestive of a neuroendocrine tumor (Figures 4A-4B). The histopathology of the lymph nodes revealed metastatic deposits of neuroendocrine tumors in lymph nodes (Figures 4C-4D).

Immunohistochemistry (IHC) with synaptophysin and chromogranin was positive. The Ki-67 index was less than 2%. The histomorphological and IHC features were consistent with a well-differentiated neuroendocrine tumor (grade 1).

The patient was initially planned for a Whipple procedure. However, during the intraoperative examination, it was decided to defer the Whipple procedure. Despite the tumor size and involvement of lymph nodes, it was found that the tumor could be easily enucleated with the necessary margin.

**Figure 5:**
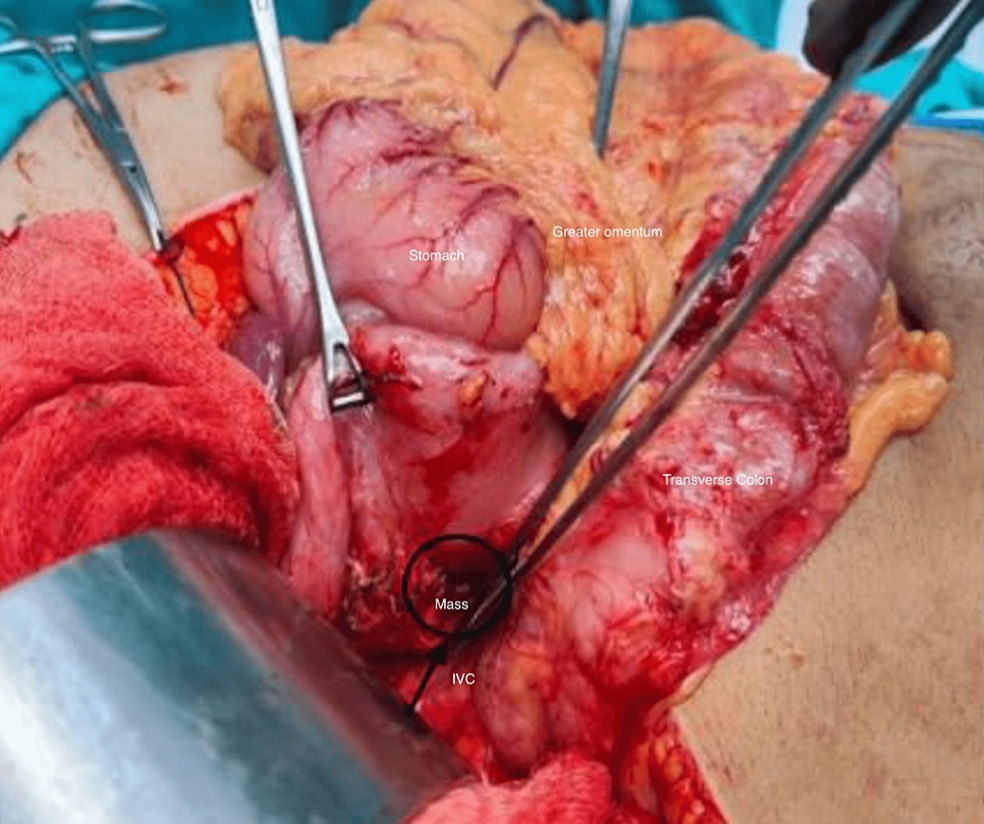
Intraoperative picture of the tumor being dissected free from the duodenum and the tumor after resection.

The intraoperative findings revealed a round, well-capsulated mass measuring approximately 4 cm x 4 cm in size. The mass was located posterior to the uncinate process and positioned below the second part of the duodenum, compressing the IVC. The mass could be separated from the IVC easily due to the presence of clear planes and a capsule (Figure 5).

**Figure 6:**
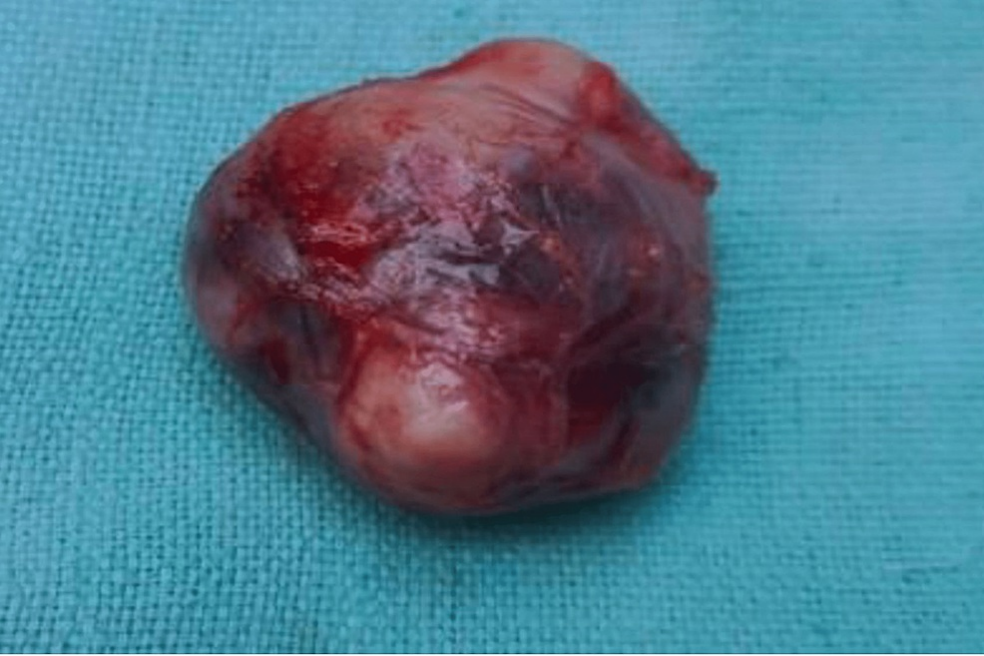
Tumor specimen after resection.

The mass was meticulously separated from the uncinate process and duodenum causing any damage to either structure. Additionally, the mass was found to be abutting the central pancreatic duct, but it could be carefully dissected away because of its encapsulated configuration, which provided a clear plane for enucleation. Two other lymph nodes present superior to the mass of size 1 cm × 1 cm were also removed. Based on these intraoperative findings, the surgical procedure was modified, and EL was performed (Figure 6). Later in the resected specimen, the biopsy findings were confirmed and the margins were free of tumor. The lymph node showed tumor deposits within. The post-op period was uneventful, and the patient was discharged on the sixth postoperative day. Considering the presence of deposits in her lymph nodes, it was decided to keep the patient on follow-up with regular repeat investigations to detect any recurrence at an early stage. She was advised to undergo surveillance for potential recurrence and advised to follow up accordingly. Between 12 weeks and 12 months post-surgery, the patient was advised to undergo a general health check-up along with specific biochemical marker tests. Additionally, a multiphasic CECT or MRI, along with a chest X-ray, was recommended. These follow-up investigations were advised to be repeated every six to 12 months for 10 years to monitor for any signs of recurrence.

## Discussion

pNETs are a group of rare, heterogenous neoplasm that arise from pluripotent stem cells in the pancreatic ductal/acinar system and produce hormones. Of all the patients, 5% have underlying familial syndrome. The molecular biology recognized in these tumors shows mutations in well-differentiated pNETs such as multiple endocrine neoplasia (MEN) in 44%, DAXX/ATRX in 43%, and mTOR in 15%. The most common mutations noted in poorly differentiated pNETs are P53 (95%), RB (74%), and Bcl2 [3,4]. Surgical options for pNETs are as follows: surgical consideration is preferred in primary tumors for functional, symptomatic pNETs, isolated G1 tumors, or patients with grade 2 pNETs with size >2 cm [4]. In patients with metastatic disease, where it is possible to resect all visible metastases, hepatic debulking is done for those with symptomatically advanced disease (with only liver metastasis) and only if approximately 80% of the liver disease is resectable. In nonfunctional pNETs, surgical resection is indicated in tumors >2 cm due to their metastatic potential. However, the optimal management of nonfunctioning pNETs <2 cm remains a matter of discussion [4].

The specifications for surgical indications in pNETs enucleation are recommended for small (<2 cm) superficial tumors, relatively close to the surface of pancreatic parenchyma without a connection or close relation to the pancreatic duct or at least 2 to 3 mm away from it (as was seen in our case). Lymphadenectomy is not performed in these tumors. Pancreatic resection or PD is indicated for tumors in the head of the pancreas. A pylorus-preserving PD (PPPD) is preferable; distal pancreatectomy (DP) is preferred for tumors in the body or tail. Splenectomy is done for larger tumors that invade the splenic vein [1,2,4].

Several studies have compared the outcomes of EL and PD for pNETs. In their research, Chen et al. concluded that enucleation was a wise choice in patients with grade 1 pNETs and a tumor diameter <4 cm if the cancer was located over 3 mm from the pancreatic duct [1]. In their research, Kabir et al. compared minimal invasive versus open enucleation. Compared with six other studies, they concluded that minimal invasive enucleation was better than open enucleation for pancreatic tumors [2]. A retrospective survey by Gaujoux et al. also concluded that EL was associated with a lower rate of complications and shorter hospital stays than PD but with a higher rate of local recurrence. However, overall survival between the two groups remained the same [4]. So, the controversy persists. Jilesen et al. studied 205 cases of postoperative complications to find that incidence of exocrine and endocrine insufficiency was significantly higher after PD (55% and 19%) compared to the tumor enucleation and DP (5% and 7% vs. 8% and 13%) [5]. In 2020, Heidsma et al. with propensity score matching (PSM) analysis found that clinically significant pancreatic fistulae incidence was higher after EN [6]. Shen et al. performed a systematic review and concluded that enucleation, compared to standard surgical resection, was associated with better clinical outcomes except for higher rates of pancreatic fistulae and, therefore, might be considered for selected pancreatic neoplasms [7]. In our case, enucleation was performed because it was away from the central pancreatic duct and the size was <4 cm, which supported the study by Chen et al. [1].

## Conclusions

In conclusion, EL and PD/DP are viable surgical approaches for treating pNETs. EL is associated with a lower risk of complications and shorter hospital stays but a higher risk of tumor recurrence, fistula formation, and reoperation than PD. The choice of surgical approach for pNETs should be individualized. Overall, the choice of surgical procedure for pNETs should be made on a case-by-case basis for the individual patient, considering the size and location of the tumor, the patient's overall health, and the surgical team's expertise.

## References

[1] Chen J, Yang Y, Liu Y, Kan H (2021). Prognosis analysis of patients with pancreatic neuroendocrine tumors after surgical resection and the application of enucleation. World J Surg Oncol.

[2] Kabir T, Tan ZZ, Syn N, Chung AY, Ooi LL, Goh BK (2019). Minimally-invasive versus open enucleation for pancreatic tumours: a propensity-score adjusted analysis. Ann Hepatobiliary Pancreat Surg.

[3] Anderson CW, Bennett JJ (2016). Clinical presentation and diagnosis of pancreatic neuroendocrine tumors. Surg Oncol Clin N Am.

[4] Gaujoux S, Partelli S, Maire F (2013). Observational study of natural history of small sporadic nonfunctioning pancreatic neuroendocrine tumors. J Clin Endocrinol Metab.

[5] Jilesen AP, van Eijck CH, Busch OR, van Gulik TM, Gouma DJ, van Dijkum EJ (2016). Postoperative outcomes of enucleation and standard resections in patients with a pancreatic neuroendocrine tumor. World J Surg.

[6] Heidsma CM, Tsilimigras DI, van Dieren S (2021). Indications and outcomes of enucleation versus formal pancreatectomy for pancreatic neuroendocrine tumors. HPB (Oxford).

[7] Shen X, Yang X (2021). Comparison of outcomes of enucleation vs. standard surgical resection for pancreatic neoplasms: a systematic review and meta-analysis. Front Surg.

